# Functional genomics reveals increases in cholesterol biosynthetic genes and highly unsaturated fatty acid biosynthesis after dietary substitution of fish oil with vegetable oils in Atlantic salmon (*Salmo salar*)

**DOI:** 10.1186/1471-2164-9-299

**Published:** 2008-06-24

**Authors:** Michael J Leaver, Laure AN Villeneuve, Alex Obach, Linda Jensen, James E Bron, Douglas R Tocher, John B Taggart

**Affiliations:** 1 Institute of Aquaculture, University of Stirling, Stirling FK9 4LA, UK; 2 Skretting Aquaculture Research Centre (ARC), P.O. Box 48, N-4001 Stavanger, Norway

## Abstract

**Background:**

There is an increasing drive to replace fish oil (FO) in finfish aquaculture diets with vegetable oils (VO), driven by the short supply of FO derived from wild fish stocks. However, little is known of the consequences for fish health after such substitution. The effect of dietary VO on hepatic gene expression, lipid composition and growth was determined in Atlantic salmon (*Salmo salar*), using a combination of cDNA microarray, lipid, and biochemical analysis. FO was replaced with VO, added to diets as rapeseed (RO), soybean (SO) or linseed (LO) oils.

**Results:**

Dietary VO had no major effect on growth of the fish, but increased the whole fish protein contents and tended to decrease whole fish lipid content, thus increasing the protein:lipid ratio. Expression levels of genes of the highly unsaturated fatty acid (HUFA) and cholesterol biosynthetic pathways were increased in all vegetable oil diets as was SREBP2, a master transcriptional regulator of these pathways. Other genes whose expression was increased by feeding VO included those of NADPH generation, lipid transport, peroxisomal fatty acid oxidation, a marker of intracellular lipid accumulation, and protein and RNA processing. Consistent with these results, HUFA biosynthesis, hepatic β-oxidation activity and enzymic NADPH production were changed by VO, and there was a trend for increased hepatic lipid in LO and SO diets. Tissue cholesterol levels in VO fed fish were the same as animals fed FO, whereas fatty acid composition of the tissues largely reflected those of the diets and was marked by enrichment of 18 carbon fatty acids and reductions in 20 and 22 carbon HUFA.

**Conclusion:**

This combined gene expression, compositional and metabolic study demonstrates that major lipid metabolic effects occur after replacing FO with VO in salmon diets. These effects are most likely mediated by SREBP2, which responds to reductions in dietary cholesterol. These changes are sufficient to maintain whole body cholesterol levels but not HUFA levels.

## Background

FO has been, and still remains, the principal lipid source for fish diets in intensive aquaculture[1]. Because the wild fisheries, from which FO is obtained, are currently fished to the maximal sustainable levels there is pressure to utilise alternative lipid sources in aquaculture diets. Currently the most practical alternatives are VOs and recent studies have demonstrated that VO can be used to replace up to 75% of FO without significant effects on growth in Atlantic salmon [2]. However the effects of VO-based diets on normal metabolism and physiology and ultimately fish health and welfare are not yet understood. A variety of potentially deleterious effects have been reported in VO-fed fish. These include cardiac lesions [3], liver histopathology [4], compromised immune function [5] and disruption of intestinal function [6-9]. There is good reason to suspect that feeding of VO may have a major impact on fish physiology since VO differ considerably in composition compared to FO. For example VOs are rich in shorter chain, C_18 _polyunsaturated fatty acids (PUFA) and devoid of n-3HUFA. Indeed, fatty acid compositions in fish, including salmon, fed VO are characterised by increased levels of C_18 _PUFA and decreased levels of n-3HUFA, which could compromise their nutritional value to the human consumer [2,10].

Massively parallel gene expression profiling technologies such as cDNA or oligonucleotide microarrays are powerful tools for discovering genes which change their tissue or cellular expression levels in response to changed conditions and thence enable the physiological mechanisms underlying such changes to be elucidated. Until recently there have been few such resources for commercially important fish species. The recent development of high density cDNA microarrays for Atlantic salmon (GRASP; TRAITS-SGP; [11,12], the most commercially valuable farmed fish species in Europe and the Americas, has opened the way for fundamental studies on diet-gene interactions and promises to greatly advance understanding of fish nutrition.

The aim of this study was to discover mechanisms for physiological adaptation to VO-based diets in fish. This was achieved by measuring the effects on hepatic gene expression using a high density cDNA microarray, and by complementary biochemical and compositional assays of Atlantic salmon smolts fed diets in which 100% of the FO was replaced with three VOs, rapeseed oil (RO), linseed oil (LO) or soybean oil (SO).

## Results

### Growth and biometry

Slightly, but significantly, lower final weights were obtained with fish fed the LO diets compared to fish fed FO (Table 1). However, no effect of dietary oil was observed in the specific growth rate (SGR) obtained for individually pit-tagged fish. Dietary VOs had no effect on feed efficiency as measured by feed conversion ratio (FCR), hepato-somatic index (HSI) or viscero-somatic index (VSI) or condition factor (Table 1).

**Table 1:** Growth, biometric parameters and proximate analyses for Atlantic salmon (*Salmo salar*) fed the experimental diets for 16 weeks

	**FO**	**LO**	**RO**	**SO**
Initial weight (g)	132.0 ± 12.3	132.4 ± 12.6	132.1 ± 12.8	131.3 ± 12.2
Final weight (g)	434.5 ± 64.1^ab^	412.9 ± 61.8^c^	447.4 ± 75.6^a^	419.7 ± 67.8^bc^
SGR	1.03	0.99	1.06	1.01
SGR (Pit-tags)	1.09 ± 0.14	1.07 ± 0.11	1.16 ± 0.10	1.1 ± 0.13
FCR	0.72	0.75	0.71	0.71
VSI	8.60 ± 0.70	8.92 ± 0.86	8.65 ± 0.72	9.06 ± 0.94
HSI	1.19 ± 0.14	1.10 ± 0.16	1.13 ± 0.15	1.14 ± 0.12
CF initial	1.05 ± 0.07	1.06 ± 0.05	1.04 ± 0.06	1.04 ± 0.07
CF final	1.24 ± 0.11	1.25 ± 0.10	1.27 ± 0.12	1.24 ± 0.12
Moisture	65.50 ± 0.80	66.80 ± 1.80	66.50 ± 0.60	68.10 ± 0.80
Protein	51.00 ± 0.50^b^	55.10 ± 1.70^a^	53.90 ± 1.30^a^	55.60 ± 0.30^a^
Lipid	40.50 ± 0.20^a^	35.80 ± 2.50^b^	38.30 ± 1.50^ab^	37.10 ± 0.20^ab^
Ash	5.70 ± 0.10^b^	6.30 ± 0.30^a^	5.90 ± 0.20^ab^	6.30 ± 0.20^a^
P/L Ratio	1.28 ± 0.02^b^	1.40 ± 0.14^ab^	1.32 ± 0.08^ab^	1.49 ± 0.01^a^

Initial and final weights (n = 250), SGR (pit-tags) (n = 20), VSI (n = 12), HSI (n = 12) and CF (n = 12) Moisture, Protein, Lipid, Ash (n = 3) are all means ± SD. Significance of differences between means were determined by one -way ANOVA followed, where appropriate, by Tukey's multiple comparison test as described in the Materials and Methods. Values within a row with a different superscript letter are significantly different (P ≤ 0.05). CF, condition factor = 100 × [(body weight (g)/body length (cm)]3; FCR, feed conversion ratio = feed consumed (kg)/weight gain (kg); HSI, hepato-somatic index = 100 × (liver weight × body weight-1); SGR, specific growth rate (%/day) = 100 × [(ln weight final – ln weight initial) × days-1]; VSI, viscero-somatic index = 100 × (carcass weight × body weight-1). P/L, Protein:Lipid ratio.

### Proximate, lipid and fatty acid compositions

All the VO diets significantly increased the relative percentage of protein in the whole fish compared to fish fed the FO diet (Table 1). There was also a tendency for the VO diets to decrease whole body lipid, and increase the protein:lipid ratio although these parameters were only significant in fish fed LO (Table 1). Liver total lipid content was not significantly affected by dietary VOs, but there was a trend towards increased free fatty acids in fish fed LO and increased total neutral lipid and triacylglycerol in fish fed SO and LO (Table 2). Dietary VO had only a few significant effects on the lipid class composition of liver with the most obvious being fish fed LO and SO showing reduced proportions of phosphatidylethanolamine compared to fish fed FO (Table 2). Differences in phosphatidylcholine levels were also observed between RO and LO, although none of the VO diets showed significant differences compared to FO. Increases in total unknown neutral lipid were also apparent in RO and SO fed fish compared to FO. In general the fatty acid compositions of liver and flesh total lipid (Table 3) reflected the fatty acid compositions of the diets. Thus fish fed the FO diet were characterised by high levels of 16:0, 20:5n-3 (eicosapentaenoic acid; EPA) and 22:6n-3 (docosahexaenoic acid; DHA), with 20:4n-6 (arachidonic acid; ARA) as the major n-6 fatty acid. Feeding the VOs resulted in increased proportions of 18:1n-9 and total monoenes, 18:2n-6 and total n-6PUFA, and 18:3n-3, and decreased the proportions of 16:0 and total saturated fatty acids, ARA, EPA and DHA. Specifically, the major fatty acids in fish fed RO, SO and LO were 18:1n-9, 18:2n-6 and 18:3n-3, respectively. Fish fed the vegetable oils also showed increased proportions of the metabolites of these fatty acids, namely 20:3n-3 and 20:4n-3 in fish fed LO, 20:2n-6 and 20:3n-6 in fish fed SO, and 20:1n-9 in fish fed RO.

**Table 2:** Lipid class composition of liver of Atlantic salmon (*Salmo salar*) fed various dietary oils

Lipid class	**FO**	**LO**	**RO**	**SO**
Total lipid	4.2 ± 0.2	4.8 ± 0.5	4.4 ± 0.5	4.8 ± 0.8
Phosphatidylcholine	17.7 ± 4.0^ab^	13.3 ± 0.5^b^	19.0 ± 1.6^a^	14.5 ± 0.8^ab^
Phosphatidylethanolamine	10.1 ± 1.5^a^	7.7 ± 1.2^b^	9.4 ± 0.7^ab^	7.8 ± 0.6^b^
Phosphatidylserine	2.6 ± 1.3	3.1 ± 0.5	3.7 ± 0.4	3.1 ± 0.5
Phosphatidylinositol	3.2 ± 1.4	2.8 ± 0.8	4.0 ± 0.3	3.4 ± 0.5
PG/CL	3.2 ± 0.9	2.2 ± 1.1	2.6 ± 0.3	1.8 ± 0.4
Sphingomyelin	1.8 ± 0.3	1.4 ± 0.3	1.8 ± 0.2	1.4 ± 0.3
Lyso-PC	2.8 ± 0.2	2.7 ± 0.4	2.2 ± 0.5	1.9 ± 0.4
Unknown polar lipid	2.0 ± 0.3	2.5 ± 0.2	3.2 ± 0.2	2.8 ± 0.3
Total polar	43.4 ± 6.9^ab^	35.7 ± 0.6^b^	45.8 ± 3.9^a^	36.8 ± 3.2^ab^
Total neutral	56.6 ± 6.9^ab^	64.3 ± 0.6^a^	54.2 ± 3.9^b^	63.2 ± 3.2^ab^
Cholesterol	15.9 ± 0.9	15.7 ± 1.0	16.0 ± 0.8	15.3 ± 0.5
Triacylglycerol	24.8 ± 7.8	29.4 ± 2.2	23.1 ± 3.5	28.3 ± 2.4
Free fatty acid	7.7 ± 2.3	12.1 ± 1.8	8.0 ± 4.0	10.4 ± 0.8
Steryl ester	6.9 ± 2.1	5.6 ± 2.2	3.8 ± 1.3	5.6 ± 1.9
Unknown neutral lipid	1.3 ± 0.9^b^	1.4 ± 0.8^b^	3.3 ± 0.5^a^	3.6 ± 0.6^a^

Values are means ± SD of 4 samples each of tissue pooled from 3 fish. Significance of differences between means were determined by one-way ANOVA followed, where appropriate, by Tukeys multiple comparison post hoc test. Values within a row with a different superscript letter are significantly different (P ≤ 0.05). CL, cardiolipin; Lyso-PC, lysophosphatidylcholine; PG, phosphatidylglycerol. All values are expressed as percentage total lipid, except total lipid which is expressed as percentage of wet weight.

**Table 3:** Fatty acid composition (percentage of weight) of total lipid from livers of Atlantic salmon (*Salmo salar*) fed different dietary oils

	**FO**	**LO**	**RO**	**SO**
14:0	2.7 ± 0.4^a^	0.6 ± 0.0^b^	1.0 ± 0.1^b^	0.6 ± 0.0^b^
16:0	16.7 ± 0.8^a^	10.0 ± 0.8^b^	12.1 ± 1.0^b^	11.4 ± 1.3^b^
18:0	5.9 ± 0.4^a^	6.0 ± 0.4^a^	4.4 ± 0.2^b^	6.1 ± 0.4^a^
Total saturated^1^	25.8 ± 0.5^a^	16.9 ± 1.0^b^	17.9 ± 0.9^b^	18.4 ± 1.2^b^
16:1^2^	3.7 ± 0.6^a^	1.0 ± 0.1^c^	1.8 ± 0.1^b^	0.9 ± 0.1^c^
18:1n-9	9.3 ± 1.1^c^	18.4 ± 1.7^b^	29.1 ± 2.0^a^	18.4 ± 2.2^b^
18:1n-7	2.9 ± 0.1^a^	1.1 ± 0.1^d^	2.4 ± 0.1^b^	1.6 ± 0.1^c^
20:1^3^	2.5 ± 0.4^b^	2.0 ± 0.1^b^	3.8 ± 0.4^a^	2.2 ± 0.2^b^
22:1^4^	1.0 ± 0.4	0.8 ± 0.1	1.0 ± 0.2	0.6 ± 0.1
24:1n-9	1.0 ± 0.1^a^	0.6 ± 0.1^b^	0.8 ± 0.1^ab^	0.6 ± 0.1^b^
Total monoenes	20.3 ± 2.6^b^	24.0 ± 1.9^b^	39.0 ± 2.5^a^	24.3 ± 2.4^b^
18:2n-6	1.9 ± 0.4^c^	9.6 ± 0.3^b^	8.6 ± 0.3^b^	26.4 ± 1.8^a^
20:2n-6	0.5 ± 0.0^c^	1.4 ± 0.1^b^	1.6 ± 0.1^b^	4.3 ± 0.2^a^
20:3n-6	0.3 ± 0.1^d^	0.6 ± 0.1^c^	1.1 ± 0.1^b^	2.5 ± 0.1^a^
20:4n-6	2.8 ± 0.4^a^	0.7 ± 0.1^c^	1.4 ± 0.2^b^	1.6 ± 0.2^b^
Total n-6 PUFA^5^	6.4 ± 0.1^c^	12.5 ± 0.3^b^	13.1 ± 0.1^b^	35.3 ± 1.7^a^
18:3n-3	0.6 ± 0.2^c^	20.3 ± 0.9^a^	2.4 ± 0.2^b^	2.3 ± 0.1^b^
18:4n-3	0.6 ± 0.3^a^	0.8 ± 0.1^a^	0.3 ± 0.1^b^	0.2 ± 0.0^b^
20:3n-3	0.2 ± 0.1^b^	3.2 ± 0.3^a^	0.4 ± 0.1^b^	0.3 ± 0.0^b^
20:4n-3	1.2 ± 0.3^b^	3.0 ± 0.0^a^	1.0 ± 0.1^b^	0.6 ± 0.1^c^
20:5n-3	10.7 ± 0.3^a^	4.0 ± 0.4^b^	4.8 ± 0.4^b^	2.6 ± 0.6^c^
22:5n-3	3.6 ± 0.0^a^	1.0 ± 0.1^c^	1.4 ± 0.1^b^	0.8 ± 0.2^c^
22:6n-3	30.0 ± 2.7^a^	14.3 ± 1.6^c^	19.6 ± 1.8^b^	15.2 ± 2.3^c^
Total n-3 PUFA	46.9 ± 2.2^a^	46.6 ± 1.7^a^	30.0 ± 1.8^b^	22.0 ± 3.0^c^
Total PUFA	54.0 ± 2.2^b^	59.1 ± 1.6^a^	43.1 ± 1.7^c^	57.3 ± 1.5^ab^

Values are means ± SD of 4 samples each of tissue pooled from 3 fish. Significance of differences between means were determined by one-way ANOVA followed, where appropriate, by Tukey's multiple comparison test as described in the Materials and Methods. Values within a row with a different superscript letter are significantly different (P ≤ 0.05). 1, contains 15:0, 20:0 and 22:0, present in some samples at up to 0.4%; 2, predominantly n-7 isomer; 3, predominantly n-9 isomer; 4, predominantly n-11 isomer; 5, totals include 18:3n-6, 22:4n-6 and 22:5n-6 present in some samples at up to 0.5%.

### Liver enzyme activities

**Figure 1:**
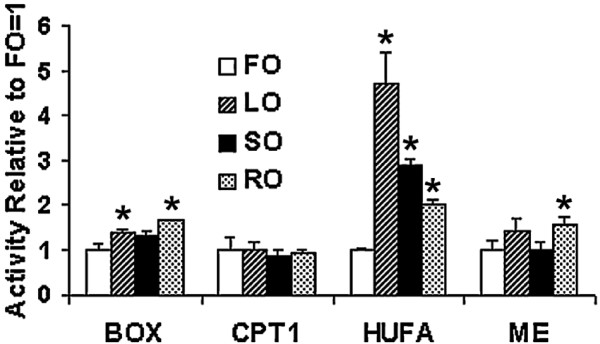
**Expression of various enzyme activities in vegetable oil fed Atlantic salmon**. Results are expressed relative to fish oil. Those which are significantly different from fish oil (p ≤ 0.05 t-test) are indicated with an asterisk. BOX, total palmitoyl CoA beta-oxidation; CPT1, carnitine palmitoyl CoA transferase; HUFA, highly unsaturated fatty acid synthesis; ME, malic enzyme. Specific enzyme activities for control fish are: BOX, 33.6 ± 4.7 pmol palmitoyl-CoA oxidised/min/mg protein; CPT1, 56.7 ± 14.8 pmol palmitoyl-carnitine produced/min/mg protein; HUFA, 2.88 ± 0.1 pmol linolenic acid desaturation products/h/mg protein; ME, 69.8 ± 15.2 nmol malate oxidised/min/mg protein.

No effect of diet was detected on the activity of liver carnitine palmitoyl transferase 1 (CPT1) activity, although fatty acid β-oxidation was increased in LO and RO fed fish. HUFA synthesis was increased in liver microsomes from fish fed all the VOs compared to fish fed FO. The activity of malic enzyme (ME) was increased in fish fed the RO diet in comparison to fish fed FO (Fig. 1).

### Microarray gene expression profiling

Comparison of each experimental diet to FO identified 428, 350 and 434 features from RO, LO and SO respectively which were significantly different (p ≤ 0.05). These lists from each VO were then compared and features which were significantly changed relative to FO in two or more VO diets (T-test, P ≤ 0.05) were retained. This resulted in a list of 132 features. Features were then eliminated which were changed in different directions in different VO diets, resulting in a list of 128 features, 88 of which could be tentatively identified according to BLAST results. These genes whose expression was changed, and whose identity could be indicated from BLAST similarity, in two or more VO diets compared to FO, are listed in Table 4.

**Table 4:** Genes in liver changed in two or more vegetable oil diets compared to salmon fed fish oil

**PROCESS**	**ACC. NO**.	**LO**	**RO**	**SO**	**EST-BLASTX OR EST-TC-BLASTX**
HUFA BIOSYNTHESIS	DW588445	**4.131**	**6.1**	**3.529**	(Q9DEX7) Delta-5/delta-6 fatty acid desaturase
	EG354827	**3.814**	**3.864**	0.685	(AAL82631) Salmon delta-5 fatty acyl desaturase, TC51151
	EG647320	**4.286**	**3.37**	1.909	(Q9DEX7) Delta-5/delta-6 fatty acid desaturase
	AAL82631	**5.624**	**11.29**	**8.027**	(Q9DEX7) Delta-5/delta-6 fatty acid desaturase
	AAL82631	**1.84**	**3.657**	**2.932**	(Q9DEX7) Delta-5/delta-6 fatty acid desaturase
	AAR21624	**2.564**	**4.248**	2.688	(Q9DEX7) Delta-5/delta-6 fatty acid desaturase

CHOLESTEROL AND ISOPRENOID BOSYNTHESIS	CK875291	**13.61**	12.23	**13.86**	(O35760) Isopentenyl-diphosphate delta-isomerase 1
	CO471940	**6.282**	**8.078**	**6.958**	(Q4R4W5) Isopentenyl-diphosphate delta-isomerase 1
	EG647637	**14.11**	2.921	**20.59**	(P52019) squalene epoxidase
	CK874989	**4.679**	**4.488**	3.376	(O88822) Lathosterol oxidase
	CK881623	1.132	**0.762**	**0.764**	(O42563) Cytochrome P450 3A27

LIPID METABOLISM AND TRANSPORT	NAN	**1.494**	1.881	**1.613**	(CAA57449) apolipoprotein B [Salmo salar], TC40920
	AM042514	1.085	**1.84**	**1.618**	(Q96BZ4) PLD4 phospholipase D family, member 4, TC47857
	DW589827	**1.403**	0.968	**1.27**	(P59837) Retinol dehydrogenase 12
	BF228485	**0.841**	**0.752**	1.228	(Q9R182) Angiopoietin-related protein 3
	NAN.	**0.489**	**0.709**	**0.652**	(Q9BYV7) BCDO2 Beta-carotene dioxygenase 2
	AJ416951	**0.541**	**0.696**	0.799	(O88275) Peroxisome proliferator-activated receptor gamma
	AM402843	**0.362**	0.67	**0.441**	(Q5TZ29) Novel protein similar to vertebrate apolipoprotein B

CARBOHYDRATE METABOLISM	CK880626	1.093	**1.816**	**2.013**	(P05370) G6PD Glucose-6-phosphate 1-dehydrogenase
	AM083390	**2.685**	1.291	**1.873**	(Q5MIB6) Glycogen phosphorylase
	NAN	**2.316**	**1.609**	1.256	(Q04760) GLO1 glyoxalase 1, TC39739
	EG355381	**0.47**	**0.659**	0.7	(P15121) AKR1B1 aldo-keto reductase 1B1, TC40780

MITOCHONDRIAL FUNCTION	DW589236	**8.96**	**2.524**	1.84	(Q96EL2) MRPS24 mitochondrial ribosomal protein S24, TC34551
	CK891719	**3.013**	**2.495**	**2.212**	(Q99595) Mitochondrial import inner membrane translocase Tim17 A
	NAN	2.067	**1.455**	**1.582**	(Q9EQ20) aldh6a1 aldehyde dehydrogenase 6A1, TC37457
	CK881059	0.97	**0.515**	**0.587**	(Q9H2U2) Inorganic pyrophosphatase 2, mitochondrial
	EG649042	2.754	**0.644**	**0.763**	(P16036) Phosphate carrier protein, mitochondrial

PROTEIN PROCESSING AND TURNOVER	CK898137	2.407	**1.258**	**1.377**	(Q5ZIJ9) Ubiquitin ligase protein MIB2
	CK878932	**2.384**	1.532	**1.599**	(P03974) Transitional endoplasmic reticulum ATPase
	BM414061	**5.083**	**5.299**	**5.287**	(P97571) Calpain-1 catalytic subunit
	CK893924	**1.906**	**1.819**	2.368	(Q99614) Tetratricopeptide repeat protein 1
	DW591059	**1.6**	1.198	**1.72**	(ABQ57500) small heat shock protein HSPB9, TC57595
	AM397488	**0.357**	0.941	**0.517**	(P45878) FK506-binding protein 2, TC38192

RNA PROCESSING	CK888026	1.133	**1.293**	**1.791**	(P09651) heterogeneous nuclear ribonucleoprotein A1, TC61954
	CK892099	**2.289**	1.389	**1.697**	(P28048) La ribonucleoprotein A
	DW588498	1.629	**1.995**	**2.155**	(Q9D198) syf2 SYF2 homolog, RNA splicing factor, TC34249
	CK891976	**1.222**	1.093	**1.373**	(Q6PDM2) sfrs1 splicing factor, arginine/serine-rich 1, DY729372
	DW589408	**0.538**	**0.625**	0.56	(Q9D0E1) heterogeneous nuclear ribonucleoprotein M, TC35947
	CK884048	1.354	**0.792**	**0.874**	(P62317) Small nuclear ribonucleoprotein Sm D2
	AJ425602	**0.526**	**0.481**	0.578	(Q5RF83) Cold-inducible RNA-binding protein

SIGNAL TRANSDUCTION	CK898218	2.04	**1.571**	**1.642**	(Q6PH57) Guanine nucleotide-binding protein G(I)/G(S)/G(T) beta1
	CK881679	**2.84**	1.823	**2.093**	(Q8JGT0) Casein kinase I alpha, TC40170
	NAN	**0.531**	**0.637**	0.812	(Q8BXH3) gucy1b2 guanylate cyclase 1, soluble, beta 2, TC44944
	CK879754	**0.333**	0.627	**0.428**	(Q14511) Enhancer of filamentation 1
	CK877929	0.877	**0.528**	**0.416**	(Q2M5E4) RGS21 regulator of G-protein signaling 21, TC38138
	CK884662	**0.258**	**0.521**	**0.539**	(P62142) Serine/threonine protein phosphatase PP1b catalytic
	AM049833	**0.603**	0.862	**0.689**	(P62155) Calmodulin
	BF228513	0.402	**0.506**	**0.682**	(Q8CFJ8) par-6 partitioning defective 6 homolog gamma, BF228513

TRANSCRIPTION	CN181085	1.588	**1.189**	**1.597**	(Q8BQ46) TAF15 RNA polymerase II, TC53593
	EG648659	**1.637**	**1.396**	1.09	(Q6GPQ6) Endothelial differentiation-related factor 1 homolog
	AM402809	**1.363**	**1.469**	1.242	(Q8BG15) ctdspl2 CTD small phosphatase like 2
	CK898087	**0.698**	**0.627**	0.699	(Q9ERU2) Zinc finger protein 22

TRANSLATION	DW588363	1.032	**1.227**	**1.414**	(P17508) Elongation factor 1-alpha, oocyte form
	EG648663	**1.305**	1.076	**1.433**	(Q9WV70) Nucleolar complex protein 2 homolog
	NAN	**1.974**	1.225	**1.812**	(P18077) RPL35a ribosomal protein L35a, TC60182
	EG647766	**0.695**	**0.74**	0.933	(P47826) 60S acidic ribosomal protein P0
	CK886497	**0.559**	**0.688**	0.854	(P57776) Elongation factor 1-delta

EXTRACELLULAR MATRIX	CK877333	**1.554**	**2.258**	**2.234**	(Q98967) Cystatin
	CK876354	0.869	**0.671**	**0.631**	(Q5D0F2) serine protease inhibitor, Kunitz type, 2, TC52802
	AM042526	**0.534**	0.888	**0.45**	(P48307) TFPI2 tissue factor pathway inhibitor 2, TC51552
	CK885060	**0.386**	**0.419**	**0.476**	(BAB55662) collagen a3(I), TC58897
	AJ424854	**0.432**	**0.483**	0.776	(O93486) Alpha 3 type I collagen, TC43437
	AJ425530	0.191	**0.466**	**0.554**	(Q9JM06) EGF- fibulin-like extracellular matrix protein 2, TC48959

IMMUNE FUNCTION	AM042172	1.456	**0.444**	**0.472**	(AAW66975) Ig mu heavy chain secretory form, TC58483
	AM397498	0.075	**0.667**	**0.446**	(P08603) Complement factor H

ION TRANSPORT	AM402718	**0.464**	**0.561**	1.19	(Q9NYB5) solute carrier organic anion transporter 1C1, TC47786
	CK885259	0.484	**0.625**	**0.746**	(Q9YH26) Sodium/potassium-transporting ATPase alpha-1 chain
	AM402711	0.225	**0.743**	**0.369**	(Q28677) Solute carrier family 12 member 4

APOPTOSIS	CK879455	**0.432**	**0.616**	**0.587**	(O18998) Deoxyribonuclease-1

GROWTH AND PROLIFERATION	EG649002	1.157	**1.746**	**1.278**	(Q5R5D0) Cyclin-G1
	CK876621	0.74	**1.594**	**1.492**	(Q80UU6) ogfr opioid growth factor receptor, TC55122

CYTOSKELETON	EG647322	**4.417**	**4.16**	**2.259**	(P49006) MARCKS-like 1, TC40754
	DW588815	**0.518**	**0.609**	0.831	(P68372) Tubulin beta-? Chain

LYSOSOMAL	AM042088	**0.598**	0.579	**0.583**	(Q90744) Alpha-N-acetylgalactosaminidase

AA BIOSYNTH	CK890942	**0.545**	0.671	**0.727**	(A6H5Y3) mtr methionine synthase, TC52579

MUSCLE	DW591140	1.938	**1.327**	**1.268**	(P06741) MYL1 myosin, light chain 1, TC60893

RESPIRATION	EG648831	**0.467**	**0.426**	0.386	(P02142) Hemoglobin beta-1 subunit

UNKNOWN FUNCTION	EG647376	0.26	**1.808**	**1.739**	(XP_691559) hypothetical protein [Danio rerio], TC35542
	NAN	0.93	**1.98**	**1.353**	(XP_001340266) hypothetical protein [Danio rerio], TC45154
	AM042055	1.284	**1.242**	**1.75**	(Q6I9Y2) THOC7 THO complex 7 homolog, TC54560
	AM402609	**2.644**	**2.732**	**1.84**	(Q56TU0) Type-4 ice-structuring protein precursor, TC59473
	CK898312	1.492	**1.959**	**2.192**	(XP_692475) similar to CG6282-PA, isoform A, TC34514
	CK897369	**0.705**	**0.732**	**0.677**	(Q5JVS0) HABP4 hyaluronan binding protein 4, TC34600
	CK881591	**0.288**	**0.295**	**0.408**	(XP_001338932) hypothetical protein [Danio rerio], TC55348
	BM414217	**0.655**	0.969	**0.709**	(XP_708731) hypothetical protein isoform 2, TC55763

TRANSPOSON	EG647536	0.993	**1.841**	**1.305**	(XP_001341934) similar to ReO_6, retrotransposon, TC63376
	EG647893	**0.318**	0.428	**0.423**	(AAW27349) SJCHGC04625 protein, retrotransposon, TC46087
	BM414246	0.907	**0.525**	**0.54**	(ABV31711) transposase, TC43560
	CK879027	**0.76**	0.985	**0.683**	(Q04202) Transposable element Tcb2 transposase

Numbers indicate expression ratio and a significant change (VO:FO; T-test, p ≤ 0.05) is indicated by bold type. LO, linseed oil; SO, soyabean oil; RO, rapeseed oil. Accession numbers of array features are provided. TC numbers are also provided, as described in text.

Gene ontology (GO) terms for biological processes were available for 39 of the 88 features with significant BLAST similarities that were changed in VO diets. The biological process GO terms associated with these 39 genes were compared with the total GO list for the TRAITS/SGP, and tested for significant (p ≤ 0.05) overrepresentation as implemented in GeneSpring GX 7.3.1 (Agilent). Only GO categories represented by more than one gene from the final gene list were considered significant and are reported in Table 5. Overrepresented GO categories included lipid metabolism, sterol biosynthesis, isoprenoid biosynthesis, fatty acid desaturation, mRNA processing, and translational elongation. It should be noted that the current gene ontology annotations (biological process) for the TRAITS/SGP array are only available for 3921 of the 16950 features. Notably amongst those genes in the final list which were overrepresented after GO analysis, were genes showing the greatest increase in expression. These included Δ5/6 fatty acyl desaturases which function in HUFA biosynthesis (enriched GO:6636, fatty acid desaturation) and isopentenyl-diphosphate Δisomerase (IPP), lathosterol oxidase, and squalene epoxidase, all genes of the cholesterol biosynthesis pathway (enriched GO:8299, isoprenoid biosynthesis; GO6695, cholesterol biosynthesis). Levels of other genes associated with lipid metabolism (enriched GO: 6629) or transport were also changed. These included increases in apoliporotein B (apoB) and decreases in an apoB-like gene in LO and SO. Effects were seen in levels of expression of genes involved in transcription, RNA processing and translation (enriched GO:6414).

**Table 5:** Significantly enriched biological process GO categories in VO fed fish compared to FO fed fish

**GO Biological Process Category**	**Genes with GO term in input list**	**Genes with GO term in output list**	**Enrichment p-Value**
GO:6629: lipid metabolism	91	5	0.00186
GO:44255: cellular lipid metabolism	72	5	0.000643
GO:6631: fatty acid metabolism	36	3	0.00518
GO:6636: fatty acid desaturation	5	3	8.98E-06
GO:8610: lipid biosynthesis	25	2	0.025
GO:8299: isoprenoid biosynthesis	4	2	0.000571
GO:6695: cholesterol biosynthesis	4	2	0.000571
GO:6720: isoprenoid metabolism	5	2	0.000946
GO:6820: anion transport	54	3	0.0159
GO:6414: translational elongation	30	3	0.00307

Many of the remaining genes from the final list could also be grouped into distinct biological process categories, although they were not identified as such by GO analysis. Changes in carbohydrate metabolism genes included an increase in the expression of glycogen phosphorylase in all VO, and glucose 6-phosphate dehydrogenase in SO and RO. Several changes in expression levels of genes of protein degradation associated with the ubiquitin-proteosome pathway were observed, and also in the protease calpain1 which was upregulated more than five fold in all VO. Signal transduction genes were also changed and MARCKS-like 1 was increased in all VO treatments and protein phosphatase 1β decreased in all VO treatments. Genes for proteins with extracellular locations which changed in VO included the protease inhibitors cystatin, spint2 and tissue factor pathway inhibitor 2, as well as decreased expression of collagen1A3. In addition there were 44 features changed by VO for which no identity could be confirmed by BLAST (not shown).

### Quantitative PCR (QPCR)

Results for QPCR are presented in Table 6. Three genes of the cholesterol biosynthesis pathway were upregulated in VO fed fish. One of these, IPP, represented by two microarray features, was selected for QPCR, another 7-dehydrocholesterol reductase was also on the array but was not significantly changed (p = 0.07). A further two genes, mevalonate kinase (MK) and SREBP2 were selected for QPCR because they were not present on the microarray, but were identified in BLAST searches of Atlantic salmon EST collections. MK was upregulated in all three VO, whilst SREBP2 mRNA was significantly increased in LO and RO, although not in SO (p = 0.057). HMG-CoA reductase (HMGR), another gene in the cholesterol biosynthesis pathway was not changed, either on the basis of microarray data or by QPCR. Δ6 fatty-acyl desaturase mRNA was also confirmed by QPCR to increase in all VO diets. A number of other lipid metabolic genes, selected on the basis of the microarray data were also assayed by QPCR. QPCR confirmed the microarray result that carnitine palmitoyl transferase A (CPT1A) mRNA was not changed, whilst QPCR of apolipoprotein B mRNA (APOB) demonstrated upregulation in LO and RO, whereas the array results indicated upregulation in SO and LO. Three other genes (catalase, PPARβ and short chain alcohol dehydrogenase) whose expression was not different between FO and VO by array analysis were also confirmed not to be changed after QPCR. However PPARγ which showed altered expression levels on micorarray analysis was not changed as measured by QPCR.

**Table 6:** Gene expression measured by QPCR in the liver of Atlantic salmon fed vegetable oils in comparison to fish oil.

	**LO**		**SO**		**RO**	
	**Expression**	**P value**	**Expression**	**P value**	**Expression**	**P value**

**Genes changed on array**						
isopentenyl-diphosphate Δisomerase	**3.263**	**0.007**	**3.755**	**0.008**	**3.313**	**0.023**
fatty acyl Δ6 desaturase	**2.809**	**0.009**	**3.215**	**0.025**	**3.994**	**0.004**
apolipoprotein B-100	**1.535**	**0.029**	1.137	0.624	**1.416**	**0.036**
PPARγ	1.053	0.805	1.118	0.649	1.122	0.555
**Genes not on array (BLAST to ESTs)**						
Mevalonate kinase	**2.158**	**0.003**	**2.334**	**0.027**	**2.949**	**0.008**
SREBP2	**2.563**	**0.018**	2.095	0.057	**2.578**	**0.011**
adipophilin	**2.836**	**0.032**	**3.469**	**0.032**	1.338	0.459
acyl-CoA oxidase	**1.432**	**0.034**	1.227	0.343	**1.501**	**0.043**
**Reference or no change on array**						
β-actin	0.773	0.318	0.908	0.694	0.794	0.291
EF1α	1.199	0.146	1.115	0.564	1.091	0.456
AJ425111	1.079	0.714	0.988	0.939	1.153	0.338
7-dehydrocholesterol reductase	**2.624**	**0.017**	**3.319**	**0.02**	**3.826**	**0.009**
carnitine palmitoyl transferase 1A	1.125	0.411	1.173	0.403	1.099	0.477
catalase	0.919	0.617	0.972	0.892	1.1	0.495
PPARβ1	0.861	0.605	0.932	0.778	0.93	0.78
PPARβ2	0.737	0.228	0.715	0.166	0.756	0.333
HMG-CoA reductase	1.204	0.136	1.132	0.566	1.097	0.516
short chain alcohol dehydrogenase	1.014	0.936	0.806	0.343	1.035	0.865

The identity of the genes tested is indicated. Those genes which were significantly changed (n = 5 fish per treatment, p ≤ 0.05) in fish fed linseed oil (LO), soyabean oil (SO) or rapeseed oil (RO) compared to fish oil are indicated in bold. Expression numbers indicate fold change relative to fish oil.

## Discussion

Although there were some relatively small differences, no great effects of diet on growth were observed in the present trial. Nevertheless, the effects of diet on tissue fatty acid composition are very clear with VO having highly significant effects. These effects are as expected and as reported in several previous trials on salmon [10,13,14].

As a means of more fully investigating the possible effects of VO substitution, liver was chosen to undertake a gene expression profiling experiment using the recently developed TRAITS/SGP microarray for Atlantic salmon. Liver integrates a large part of the nutritional uptake from the diet and has particular roles in distributing dietary lipid, and synthesising lipid *de novo *as well as participating in detoxification and excretion pathways. The use of high density cDNA microarrays is now a well established method, and we sought to use the TRAITS/SGP array to discover genes, and indicate biochemical and physiological pathways whose expression patterns might change during adaptation to VO-based diets. In this DNA microarray hybridisation experiment, application of ANOVA indicated that 784 features were changed at p ≤ 0.05 across all diet groups. However, application of either multiple testing correction (Benjamini and Hochberg) or a post hoc test (Tukeys multiple comparison) reduced this list of features 50 fold. Nevertheless, it was apparent that amongst the features from the list generated by ANOVA which passed filters for multiple testing correction or post hoc testing, were genes for Δ5/Δ6 fatty acyl desaturases. Given that these desaturase genes were known from prior experiments to be up-regulated in VO fed salmon [15] we filtered genes according to the criteria of having expression changed in the same direction in two or more of the VO dietary conditions after individual t-tests. In support of this approach it is notable that of the 132 genes which were scored as being changed in two or more VO diets, only 4 showed expression changes in opposite directions, far less than would be expected by chance alone. We tested the validity of the list of commonly changed candidates by a combination of compositional, biochemical and independent gene expression measurement by QPCR. The overall consistency of gene expression and physiological data demonstrates the validity of our approach to interpreting the microarray results presented here.

The most consistently and highly up-regulated genes that could be identified by homology or had previously been characterised and were clearly overrepresented after GO analysis were those of the Δ5 and Δ6 fatty acyl desaturases. Desaturases are encoded by several genes in salmon and each of them were represented on the array. Every one of these showed significant up-regulation in two or more of the VO diets. QPCR confirmed that desaturase mRNAs were increased and in addition the biosynthesis of HUFA was increased in liver microsomes from VO-fed fish. However, despite this clear increase in desaturase mRNA and HUFA biosynthesis, the amount of HUFA in salmon tissue was still considerably reduced when FO was replaced by VO in the diet. These results confirm previous reports describing the expression of salmon Δ5/Δ6 desaturase, where feeding of VO has been shown to increase desaturase expression and HUFA biosynthesis [15,16].

In addition to the increase in fatty acyl desaturase expression, increases in mRNA for three enzymes (IPP, squalene epoxidase and lathosterol oxidase) of the cholesterol biosynthetic pathway were also evident from microarray data and from QPCR for IPP. To test the hypothesis that the cholesterol biosynthetic pathway was up-regulated following VO feeding the Atlantic salmon EST collections were searched for genes which are known to participate in cholesterol biosynthesis, but were not present on the TRAITS/SGP array. Two genes were identified and assayed by QPCR, MK and SREBP2. MK is known to be highly regulated by cholesterol level in mammalian liver and SREBP2 is a master transcriptional regulator of both cholesterol and HUFA biosynthesis [17]. Messenger RNA for both of these genes was increased by a similar amount to that seen for IPP. The activity of SREBP2 is controlled by cellular cholesterol status, such that reductions in membrane cholesterol trigger proteolytic cleavage of membrane bound SREBP2 and lead to its translocation to the nucleus where it drives the transcription of the genes for cholesterol and HUFA biosynthesis [17]. Of the 12 genes involved in lipid metabolism which were changed on the microarray after feeding VO, 5 are known to be regulated by SREBPs (Δ5 desaturase, Δ6 deasturase, IPP, lathosterol oxidase and squalene epoxidase) [17]. Another gene in the cholesterol biosynthesis pathway, 7-dehydrocholesterol reductase, which by QPCR was upregulated in all VO, was on present the TRAITS-SGP array, but was not present in the final output gene list. One other gene critical for cholesterol biosynthesis, HMG-CoA reductase was present on the array but remained unchanged and QPCR analysis confirmed that VO had no effect on levels of HMG CoA reductase mRNA. This is surprising since in mammals HMG-CoA reductase is regulated directly by cholesterol levels, by hormonal stimuli, and transcriptionally by SREBPs, and therefore our results suggest some differences in the regulation of this gene between fish and mammals. Nevertheless, taken together the effects on genes of the cholesterol biosynthesis pathway strongly suggests that salmon fed VO respond to reduced cholesterol levels in the diet. Although we did not measure cholesterol contents of the diets, a recent report, which quantifies cholesterol and total sterol levels in the raw ingredients used to formulate the diets, confirms the deficiency of cholesterol in VO diets [18]. Moreover, it is known that in vegetable oils the bulk of sterol is present as sitosterol, stigmasterol, campesterol or brassicasterol depending on source [19]. These plant sterols are not taken up by mammals, and indeed inhibit cholesterol uptake [20] and, although not proven, are unlikely to be taken up by fish. Therefore one of the major responses of salmon liver during adaptation to VO is up-regulation of cholesterol biosynthesis to compensate for deficiencies in the diet. Importantly, and in contrast to the situation with HUFA, lower levels of dietary cholesterol were fully compensated for, since no differences were observed in cholesterol content, in liver or in flesh between fish fed FO or VOs. From the data presented here the compensation for reduced dietary cholesterol is due to up-regulation of hepatic cholesterol biosynthetic pathways, although other pathways such as decreased bile acid synthesis might also contribute. It should be noted, however, that this experiment consisted of a 12 week trial but in a longer term trial, after feeding VO for up to 22 months, both liver cholesterol and plasma LDL cholesterol were lower compared to FO [21].

Further to the up-regulation of cholesterol biosynthesis genes, apoB-100 which is a component of low density lipoprotein (LDL) required for the transport of cholesterol from liver to other tissues, also showed increases in expression level, both from microarray and QPCR measurement. Furthermore, from the microarray results angiopoietin-related protein 3 (ANGPTL3) was down-regulated in VO fed fish. ANGPTL3 is a liver derived factor which suppresses lipid uptake from LDL by tissues and promotes fatty acid release from adipocytes [22]. Thus the reduction in ANGPTL3 observed in salmon fed VO is consistent with the increase in apoB-100 and taken together indicates increases in lipid transport to tissues from liver via LDL. Interestingly an apoB-like protein, which is related but clearly distinct from apoB-100 was down-regulated in VO fed fish, and a protein which is suggested to act as an "anti-freeze" in fish blood [23], but which resembles apoA, another lipid transport molecule, is up-regulated in all VO. Several other genes which showed changes in expression levels in VO fed fish have also been linked to lipid transport. For example cystatin, which was increased in all VO fed fish, is involved in tissue lipid uptake, suggested to be related to its role in controlling elastin and collagen breakdown in the extracellular matrix [24]. Notably in this regard there were also decreases, in two microarray features identified as a collagen subtype, COL1A3, in all VO fish.

Overall these changes indicate a change in the function of the liver with respect to cholesterol synthesis and distribution, with an increase in cholesterol biosynthesis and an increase the transport processes which distribute cholesterol from the liver to other tissues. In animals fed normal cholesterol in the diet, the majority of cholesterol in the liver, from endogenous or exogenous pathways, is transported in the bile, as cholesterol or as bile acids, and thence to the gut where, together with phosphatidylcholine, it assists in the uptake of dietary lipid. In this regard, levels of hepatic phosphatidylcholine and its precursor phosphatidylserine were one of the few lipid compositional measures to vary between dietary treatments. These compositional changes might be related to changes in SO and RO diets of phospholipase D mRNA, which encodes an enzyme which releases choline from PC. Changes in bile transport, if occurring, may have consequences for lipid digestion, uptake and transport in the intestine which might explain the accumulation of lipid droplets in intestinal cells previously been observed in salmonids fed VO [6-8]. Interestingly, similar intestinal lipid accumulation is observed in rodents with deficiencies in bile transport [25].

Also notable from the microarray data was an increase in mRNA for glycogen phosphorylase (the first step of glycogen utilisation) and also in expression of glucose-6-phosphate dehydrogenase. These systems are involved in NADPH production [26] and suggests that the requirement for reducing power for cholesterol and HUFA biosynthesis is provided by breakdown of glycogen and up-regulation of NADPH generating systems. Another enzyme of importance for biosynthetic NADPH production, ME, was also shown to have increased activity in RO fed fish.

Some reports have indicated an increase in β-oxidation in VO compared to FO fed fish [27,28]. From our microarray data we did not see increases in the expression of any genes linked to mitochondrial β-oxidation, indeed mRNAs for components of mitochondrial oxidative phosphorylation (pyrophosphates and phosphate carrier proteins) were decreased. Furthermore, QPCR and enzymic activity measurement confirmed that CPT1, an important rate limiting step in mitochondrial β-oxidation, was not significantly altered by VO feeding. However, it is known that peroxisomal β-oxidation predominates in salmon liver [29] and so the mRNA expression level of a critical enzyme of peroxisomal activity, acyl-CoA oxidase [30] was also measured. Acyl-CoA oxidase mRNA was increased in salmon fed the LO and RO diets, and this was consistent with the increased peroxisomal β-oxidation observed in fish fed LO and RO.

Of course this experiment represents a "snapshot" of gene expression 20 hours after final feeding and in liver only. At different prandial states and in different tissues the expression of genes for metabolic enzymes may be different. For example, shortly after feeding, when energy is in abundance, it is possible that the pentose phosphate pathway might be fuelled by gluconeogensis rather than glycogen breakdown, and these differences between FO and VO diets may be masked. With regard to fatty acid β-oxidation, sea bream exhibit differences in mitochondrial and peroxisomal activity depending on whether they are measured at 6 or 24 h after feeding [31].

The microarray data also indicated down-regulation of PPARγ, although, after QPCR no effect of VO feeding was apparent. PPARs are critical transcriptional regulators of lipid metabolism and energy homeostasis, and are encoded by three genes in mammals known as PPARα, PPARβ and PPARγ[32]. However, in salmon at six PPAR genes have so far been discovered and they share considerable homology [33,34]. Therefore it is possible that the microarray results reflect cross-hybridisation of different, but related transcripts, although a previous microarray study has also reported a decrease in PPARγ mRNA following VO feeding in salmon [35]. In general cDNA microarrays are unlikely to be specific tools for measuring the expression of multiple closely related genes and this problem is exacerbated in Atlantic salmon which have polyploid genomes, often possessing more members within each gene family than the majority of diploid vertebrates. Similar issues been pointed out for the Δ5 and Δ6 fatty acyl desaturase genes which are 95% identical at the nucleotide level in salmon [12].

Increased lipid content, which might be an indication of health problems, in the livers of salmon fed VO has been observed previously [21,36]. In our study, although there were no significant increases in hepatic lipid, a trend for increased lipid was supported by higher TAG and free fatty acid in VO fed fish and levels of mRNA for a marker for intracellular lipid accumulation, adipophilin [37], were increased in SO and LO, supporting the idea that at least some VO diets cause increases in hepatic lipid accumulation.

Finally from the microarray results, although not directly addressed by QPCR or biochemistry, were changes in the expression of genes involved in RNA and protein processing and turnover. These changes may be a reflection of changes in cholesterol biosynthetic pathways and in lipid transport, reflecting a requirement to export cholesterol to tissues.

## Conclusion

In conclusion the present study demonstrates the utility of transcriptomic tools for studies on non-model commercially important fish species. Although, there were limitations in the statistical power of the design due largely to the inherent variability amongst recently domesticated farmed Atlantic salmon, by using appropriate filtering of microarray data, independent gene expression tests and biochemical and compositional data we were able to physiologically anchor a significant proportion of the gene expression results. This combined approach leads to the hypothesis that the major lipid metabolic effects of replacing FO with VO in salmon diets are mediated by SREBP2, a transcription factor which responds to reductions in membrane cholesterol level caused by reduced cholesterol in VO based diets. Furthermore, the application of the TRAITS/SGP microarray indicated several genes significantly altered by vegetable oils which suggest mechanisms to explain previously observed health impairment in VO fed salmon.

## Methods

### Diets and animals

Four diets (4 mm pellets) with the same basal composition but coated with four different oils were prepared at the Skretting Technology Centre, Stavanger, Norway. The diets were formulated to satisfy the nutritional requirements of salmonid fish (U.S. National Research Council 1993), and to contain 33% fat and 47% protein, consisting of fish meal (55%), corn gluten (10%), wheat (8.3%), oil (26.3%), mineral and vitamin mixes (0.2%) and Carophyl Pink^® ^(0.06%). The oils used were FO (Anchovy oil, Skretting, Stavanger, Norway), RO (Denofa AS, Fredrikstad, Norway), LO (NOBA Vetveredeling B.V., Hamburg, Germany) and SO (Denofa AS, Fredrikstad, Norway). Fatty acid and proximate compositions of the experimental diets are given in Table 7. One thousand Atlantic salmon post-smolts, average weight 132 g, were distributed randomly into four 2 × 2 m tanks (250/tank) at the Nutreco Aquaculture Research Centre, Lerang Research Station, Stavanger, Norway. In order to measure growth in individual salmon, twenty fish per tank were PIT-tagged by implanting a micro-transponder into the peritoneal cavity. The fish were conditioned to the new environment for 3 weeks before feeding the experimental diets. During the conditioning period, the fish received commercial diet containing FO (Atlantic 4 mm; Skretting). Feed intake was monitored during this period, and the trial did not start until appetite was at least 0.8% body weight. After the conditioning period, the fish were fed the experimental diets to satiation according to usual procedures at Lerang Research Station, for a period of 16 weeks. Feed intake was monitored throughout the trial and waste feed was collected from the effluent water from each tank by a wire mesh collector and dried. Feed given, waste feed and the resulting net feed intake were registered daily. The tanks were supplied with sea water at constant temperature (7.9°C ± 0.1°C). The oxygen level varied between 8 and 12 ppm, with an average of 9.9 ppm. A photoperiod of 18 h light and 6 h darkness was applied.

**Table 7:** Proximate composition. (percentage of total weight) and fatty acid compositions (percentage of total FA weight) of experimental diets

	**FO**	**LO**	**RO**	**SO**
Total Protein	46.9	46.0	48.0	47.0
Total Fat	33.2	33.2	34.3	34.1
Ash	6.7	7.6	7.0	7.8
Moisture	6.2	5.8	5.7	4.5
14:0	7.4	0.9	1.2	0.9
16:0	18.7	7.4	7.0	12.6
18:0	3.9	3.5	1.9	3.1
Total saturated^1^	30.9	11.6	10.1	16.6
16:1n-7	7.5	0.9	1.3	0.9
18:1n-9	8.8	17.4	47.2	20.8
18:1n-7	2.7	1.0	2.7	1.7
20:1^2^	3.3	1.5	2.8	1.7
22:1^3^	3.5	2.0	2.5	2.0
24:1n-9	0.5	0.2	0.3	0.2
Total monoenes	26.3	23.0	56.8	27.3
18:2 n-6	3.5	14.2	17.8	44.4
CLA (9c,11t)	0.0	0.0	0.0	0.0
CLA (10t,12c)	0.0	0.0	0.0	0.0
20:4 n-6	0.9	0.1	0.1	0.1
Total n-6PUFA^4^	4.7	14.4	18.0	44.6
18:3 n-3	1.2	44.9	8.1	5.7
18:4 n-3	3.4	0.7	0.8	0.6
20:4 n-3	0.8	0.1	0.1	0.1
20:5 n-3	14.7	2.0	2.5	2.0
22:5 n-3	1.7	0.2	0.2	0.2
22:6 n-3	16.3	2.9	3.4	2.9
Total n-3PUFA	38.1	50.8	15.1	11.5
n-6/n-3	0.12	0.28	1.19	3.89
Total PUFA	42.8	65.2	33.1	56.1

Data are means of duplicate analyses. 1, contains 15:0 and 17:0, present in some samples at up to 0.5%; 2, predominantly n-9 isomer; 3, predominantly n-11 isomer; 4, totals include 18:3n-6, 20:2n-6, 22:4n-6 and 22:5n-6 present in some samples at up to 0.5%; PUFA, polyunsaturated fatty acid.

### Sampling protocols

At the start and end of the trial, all the fish in each tank were anaesthetised with metacain (50 mg/L) and individually weighed and measured. At the end of the trial, the 20 fish per dietary treatment were sampled for compositional, enzymatic and gene expression analyses, with three whole fish frozen immediately on dry ice and subsequently stored at -20°C for whole body compositional (proximate) analyses. The other sampled fish were eviscerated and twelve were used for biometric determinations (hepato- and viscero-somatic indices) and for lipid analyses. Livers were taken from each fish, pooled in four pools of three fish each, and frozen immediately in liquid nitrogen. Samples of liver were dissected from the remaining five fish from each dietary treatment and these samples were individually used for enzymatic, microarray and quantitative PCR (QPCR) analysis. A portion of each liver was taken for enzymatic analyses and frozen in liquid nitrogen. For gene expression analysis, samples of 0.5 g of fresh tissue were rapidly disrupted in 5 ml of TriReagent using an Ultra-Turrax homogeniser (Fisher Scientific, Loughborough, U.K.), and immediately frozen in liquid nitrogen for storage prior to RNA extraction.

### Compositional analyses

Proximate analysis, consisting of moisture content and lipid and protein content, was undertaken as previously described [38]. Lipid analyses were conducted on liver from twelve (four pools of three) fish from each diet and homogenised into pooled "pates". Total lipid was extracted from diets or 1 g portions of tissue pates and separation of lipid classes was performed by high-performance thin-layer chromatography (HPTLC) and fatty acid analysis performed as described previously [38].

### Enzymatic and metabolic assays

All enzymatic and metabolic assays were carried out on the same five fish randomly selected from each dietary treatment. HUFA biosynthesis was determined in liver microsomes as described by Leaver et al., 2006. Assays of malic enzyme (ME), carnitine acyltransferase I (CPT1) and peroxisomal fatty acid β-oxidation in tissue homogenates were carried out on liver homogenates as previously described [14,38].

### Microarray gene expression profiling

Liver was selected as the tissue on which to focus gene expression studies because of its central role in the integration and control of nutritional physiology and energy homeostasis. Total RNA was extracted from the same five fish from each dietary treatment as used for enzymatic determinations. RNA was prepared from TriReagent homogenates as described in the manufacturers protocol (Sigma). Two separate RNA extracts were prepared from each sample, one for microarray probe preparation and the second for QPCR. Each biological replicate was co-hybridised in a two dye experiment with a single pooled reference sample. The pooled reference sample comprised equal amounts of RNA from each of the 20 biological samples. RNA was reverse transcribed and labelled with either Cy3 (pooled reference) or Cy5 (FO, RO, SO and LO fed fish) fluors using the FAIRPLAY II cDNA indirect labelling kit (Stratagene) according to the manufacturer's instructions. Briefly 20 μg total RNA was reverse transcribed after being primed with oligo dT, which incorporated aminoallyl-dUTP into the newly synthesised cDNA strand. The RNA template was then hydrolysed using 1 M NaOH for 15 min and neutralised with 1 M HCl. The cDNA was precipitated overnight. cDNA pellets were washed in 80% ethanol and air dried before being resuspended in 5 μL 2× coupling buffer (Stratagene FAIRPLAYII reagent). Once the cDNA had fully dissolved (after at least 30 min) 5 μL of either Cy3 or Cy5 dye was added and the samples incubated in the dark for 30 min. The Cy3 and Cy5 dyes (GE Healthcare) were dissolved in DMSO prior to being added to the coupling buffer. To remove unincorporated dye, the labelled cDNA (total volume 10 μL) was passed through a SpinEX column (Qiagen). Dye incorporation was assessed by separating 0.5 μL of the sample on a mini-agarose gel and visualising fluorescent products on a fluorescence scanner (Typhoon Trio, GE Healthcare). No microarray pre-hybridisation step was required. For hybridisation the remainder of each labelled cDNA (7–9 μL; 16–30 pmol each dye) was added to 85 μL hybridisation buffer (UltraHyb, Ambion), 10 μL poly(A)_80 _(10 mg mL^-1^; Sigma) and 5 μL ultrapure BSA (10 mg mL^-1^; Ambion). The hybridisation mixture was heated to 95°C for 3 min, then cooled to 60°C before being applied to the microarray (TRAITS/SGP, see section 2.7). Hybridisations were performed on a Gene TAC Hyb Station (Genomic Solutions) for 16 h at 45°C. Slides were then automatically washed with 2×SSC, 0.5% SDS for 10 min at 60°C; 0.2×SSC, 0.5% SDS for 10 min at 42°C; and finally 0.2 × SSC 10 min at 42°C. Following manual rinsing in isopropanol and drying by centrifugation, hybridised slides were scanned at 10 μm resolution using a Perkin Elmer ScanArray Express HT scanner. BlueFuse software (BlueGnome) was then used to identify and quantify features. Following manual spot editing to remove obvious artefactual features and fusion of duplicate spot data (BlueFuse proprietary algorithm), the resulting intensity values and quality annotations were exported into the GeneSpring GX version 7.3.1 (Agilent Technologies) analysis platform. Data transformation, normalisation and quality filtering were as follows: 1) all raw spot intensity values less than 0.01 were set to 0.01 to facilitate comparisons of transformed data; 2) a 'per spot per chip' intensity dependent (Lowess) normalisation was undertaken using software defaults (20% smoothing/cutoff 10); 3) data were filtered using a BlueFuse spot confidence value ≥ 0.2 in ≥ 8 slides and BlueFuse spot quality of ≥ 0.5 in ≥ 8 slides to filter out features which were below reasonable quality thresholds Bluefuse uses Bayesian statistical models of the signal and noise distributions on the slide to estimate the confidence that should be placed in each result. Similarly Bluefuse integrates various spot quality parameters (eg circularity, uniformity etc) to provide a spot quality assessment. This gave a final list of 11024 genes which were eligible for statistical analysis and application of ANOVA indicated that 9964 features had sufficient data for testing across all treatments. Experimental annotations complied fully with MIAME guidelines [39] and data has been submitted to the ArrayExpress databank (accession E-TABM-478; [40]).

Atlantic salmon ESTs were tentatively identified using BLASTX to search the Genbank non-redundant protein sequence database (all non-redundant GenBank CDS translations + RefSeq Proteins + PDB + SwissProt + PIR + PRF databases) applying a cut-off value of ≤ e^-10^. ESTs which were not assigned using this procedure were then compared to the Atlantic salmon DFCI Atlantic salmon Gene Index  comprising ESTs and ESTs assembled into Tentative Consensus (TC) sequences using BLASTN. TCs or ESTs showing 99.9% identity over a continuous overlap of >100 nucleotides were then used to search the Genbank non-redundant protein sequence database applying a cut-off value of ≤ e^-10^.

### Quantitative PCR (QPCR)

QPCR was performed using an RNA extract from the same five animals that had been taken for enzymic and microarray analysis. Complementary DNA (cDNA) was synthesized from total salmon hepatic RNA using a commercially available kit according the manufacturers instructions (*Reverse*-iT Max 1^st ^strand synthesis kit, ABgene). Briefly each reaction of 20 μL contained 1.5 μg of total RNA, 300 ng of random hexamers and 125 ng of anchored oligo-dT. Following cDNA synthesis at 42°C for 1 hour reactions were stopped by heating at 75°C for 10 min and cDNA diluted to 500 μL total volume with water. Real-time PCR was performed using a Quantica machine (Techne). Quantitative PCR analysis for each gene was performed in triplicate in a total volume of 20 μL containing 5 μL cDNA (equivalent to 15 ng of input RNA), 100 nM of each primer and 10 μL of Absolute QPCR SYBR Green Mix 2× (ABgene). For each target gene, forward and reverse primers were chosen from the available EST sequences by using Primer3 software[41]. Target genes were selected by consideration of microarray data and by BLAST comparison of Atlantic salmon ESTs deposited in Genbank with sequences of mammalian genes of interest. Primer pairs and EST information are provided in Table 8. Thermal cycling was initiated with incubation at 95°C for 15 min in order to activate the Thermo-Start^® ^DNA Polymerase present in the mix. After this initial step, forty-five cycles of PCR were performed. Each PCR cycle consisted of heating for 15 s at 95°C for denaturing, and then for 15 s at 60°C and 30 s at 72°C for annealing and extension. Cycle threshold (CT) values corresponded to the number of cycles at which the fluorescence emission monitored in real time exceeded the threshold limit.

**Table 8:** Genes, accession numbers and primers used for quantitative PCR

	**Acc No**	**Forward primer**	**Reverse Primer**
**Genes from Array**			
isopentenyl-diphosphate Δisomerase 1	CK875291	ACAGCCCTATGGTTATGTGTCATCTC	CAAGGTGAGGCGAATGTTTGAAC
7-dehydrocholesterol reductase	DW561983	CTTCTGGAATGAGGCATGGT	ACAGGTCCTTCTGGTGGTTG
HMG-CoA reductase	BE518590	CCTTCAGCCATGAACTGGAT	TCCTGTCCACAGGCAATGTA
Δ6 desaturase	AY458652	CCCCAGACGTTTGTGTCAG	CCTGGATTGTTGCTTTGGAT
apolipoprotein B-100	EG648437	GCTGTGGCTTTAATCCTTGC	CCATGGTGTCAGTGATCAGG
catalase	CK874978	CAACCCCCAGACTCACCTAA	GAAGGTGTGAGAGCCGTAGC
carnitine palmitoyl transferase 1A	AM230810	CCTGTACCGTGGAGACCTGT	CAGCACCTCTTTGAGGAAGG
short chain alcohol dehydrogenase	CK884238	TCTGCACACGAGAGGCATAC	GTCAGGGCAGTCACAGCATA
PPARβ1	AJ416953	GACCACCAACCCCAATGGCTCGGAT	CAGCCCATTCTCAGCCTGGCACAAG
PPARβ2	AM229303	CCCCCACCATCTTGGTGGCTCAGAC	TAGACCACTCTCTGCTTGCCACAGG
PPARγ	AJ416951	ACCCAGGAACCCAGAGTCAGCGGAC	TTTATAAAGTACTGACCCGCCGTCA
**Genes not on array (BLAST to ESTs)**			
Mevalonate kinase	DY708590	CCCTTAATCAGGGTCCCAAT	GGTGCTGGTTGATGTCAATG
SREBP2	DY733476	GACAGGCACAACACAAGGTG	CAGCAGGGGTAAGGGTAGGT
adipophilin	BM413877	GCAGACATGGAAGTCGTTGA	ATGGAAATTTGTGGCTCCAG
acyl-CoA oxidase	DW555420	AAAGCCTTCACCACATGGAC	TAGGACACGATGCCACTCAG
**Reference (no change in array ept)**			
β-actin	AF012125	ACATCAAGGAGAAGCTGTGC	GACAACGGAACCTCTCGTTA
EF1α	AF321836	CTGCCCCTCCAGGACGTTTACAA	CACCGGGCATAGCCGATTCC
unknown	AJ425111	AGCCTATGACCAACCCACTG	TGTTCACAGCTCGTTTACCG

Melting curve analysis was performed to indicate the production of a single product in these reactions. Standard curves were established for each gene by plotting CT values against the log_10 _of five different dilutions (in triplicate) of cDNA sample solutions. In addition a subset of randomly selected samples generated by each primer pair was analysed by agarose gel electrophoresis and sequencing to confirm the identity of amplicons. Real-time efficiency was determined for each gene from the slopes given by Quantsoft software, applying the equation E = 10^(-1/*slope*)^. The calculated relative expression ratio of each gene was based on the PCR efficiency (E) and CT of sample compared with control, and expressed in comparison to the reference genes, glyceraldehyde phosphate dehydrogenase (GAPDH), β-actin, and unidentified EST Acc No. AJ425111 (REST^© ^software, [42]). Statistically significant differences in gene expression between the control (FO) and samples (LO, SO and RO) were evaluated in group means by randomization tests [43] using REST^© ^software. Five thousand random allocations were applied and differences were considered to be significant at P ≤ 0.05.

### Materials

Materials for enzyme assays: [1-^14^C] Palmitoyl CoA (50–55 mCi/mmol) and [methyl-^3^H} L-carnitine (60–86 Ci/mmol) were obtained from GE Healthcare. BHT, Carnitine, coenzyme A, DTT, FAF-BSA, glutathione, HEPES, KCN, malate, N-acetylcysteine, NADH, NADP, nicotinamide, palmitoyl-CoA, perchloric acid, silver nitrate and TriReagent were obtained from Sigma.

Materials for lipid analyses: HPTLC (10 cm × 10 cm × 0.15 mm) and TLC (20 cm × 20 cm × 0.25 mm) plates, precoated with silica gel 60 (without fluorescent indicator) were obtained from Merck. All solvents were HPLC grade and were obtained from Fisher Scientific.

Microarrays: the TRAITS/SGP Atlantic salmon microarray comprised 16950 minimally redundant, duplicate cDNA clones and has been described elsewhere [12,44].

### Statistical analysis

Unless otherwise stated, all data are presented as means ± SD (n value as stated). The effects of dietary treatment on biometry, composition and enzyme activity were analysed by one-way analysis of variance (ANOVA) followed, where appropriate, by Tukey's comparison test. Percentage data and data which were identified as non-homogeneous (Bartlett's test) were subjected to arcsine transformation before analysis. Differences were regarded as significant when P ≤ 0.05. Statistical treatment of microarray and QPCR data is described within the specific Methods and Results sections.

The probability that a particular biological process GO term was enriched in the input GO list (All GO annotations in the TRAITS-GS array) compared to the output GO list (all GO annotations in the experimentally altered gene list) was calculated using a hypergeometric distribution model as implemented by GeneSpring GX 7.3.1. The GO annotation of the TRAITS-GS microarray has been described by Taggart et al., 2008 [12]

## Authors' contributions

MJL conceived and co-ordinated the study, integrated the data, performed biochemical studies and drafted the manuscript. LANV performed microarray sample preparation and hybridisations and QPCR. AO participated in experimental design, formulated diets and performed analysis of growth parameters. LJ performed dietary trial and co-ordinated sampling procedures. JEB participated in the analysis of microarray data and other statistical analyses. DRT performed lipid analyses and fatty-acyl desaturation assays. JBT participated in the co-ordination of microarray studies and experimental design. All authors read and approved the final manuscript.
